# Autophagy activated by silibinin contributes to glioma cell death via induction of oxidative stress-mediated BNIP3-dependent nuclear translocation of AIF

**DOI:** 10.1038/s41419-020-02866-3

**Published:** 2020-08-14

**Authors:** Chongcheng Wang, Chuan He, Shan Lu, Xuanzhong Wang, Lei Wang, Shipeng Liang, Xinyu Wang, Meihua Piao, Jiayue Cui, Guangfan Chi, Pengfei Ge

**Affiliations:** 1 Department of Neurosurgery, First Hospital of Jilin University, 130021 Changchun, China; 2 Research Center of Neuroscience, First Hospital of Jilin University, 130021 Changchun, China; 3 Department of Radiotherapy, Second Hospital of Jilin University, 130021 Changchun, China; 4 Department of Anesthesiology, First Hospital of Jilin University, 130021 Changchun, China; 5 Department of Histology and Embryology, College of Basic Medical Sciences, Jilin University, 130021 Changchun, China; 6 Key Laboratory of Pathobiology, Ministry of Education, Jilin University, 130021 Changchun, China

**Keywords:** CNS cancer, CNS cancer

## Abstract

Induction of lethal autophagy has become a strategy to eliminate glioma cells, but it remains elusive whether autophagy contributes to cell death via causing mitochondria damage and nuclear translocation of apoptosis inducing factor (AIF). In this study, we find that silibinin induces AIF translocation from mitochondria to nuclei in glioma cells in vitro and in vivo, which is accompanied with autophagy activation. In vitro studies reveal that blocking autophagy with 3MA, bafilomycin A1 or by knocking down ATG5 with SiRNA inhibits silibinin-induced mitochondrial accumulation of superoxide, AIF translocation from mitochondria to nuclei and glioma cell death. Mechanistically, silibinin activates autophagy through depleting ATP by suppressing glycolysis. Then, autophagy improves intracellular H_2_O_2_ via promoting p53-mediated depletion of GSH and cysteine and downregulation of xCT. The increased H_2_O_2_ promotes silibinin-induced BNIP3 upregulation and translocation to mitochondria. Knockdown of BNIP3 with SiRNA inhibits silibinin-induced mitochondrial depolarization, accumulation of mitochondrial superoxide, and AIF translocation from mitochondria to nuclei, as well as prevents glioma cell death. Furthermore, we find that the improved H_2_O_2_ reinforces silibinin-induced glycolysis dysfunction. Collectively, autophagy contributes to silibinin-induced glioma cell death via promotion of oxidative stress-mediated BNIP3-dependent nuclear translocation of AIF.

## Introduction

Glioma is an aggressive malignant brain tumor and constitutes the major causes leading to death in both pediatric and adult populations^1^. The median survival time of the patients with newly diagnosed glioma is only 14.6 months, despite these patients are treated with surgical removal of tumor followed by radiotherapy and chemotherapy with temozolomide (TMZ)^2^. As an alkylating agent, TMZ is widely used for treating primary and recurrent high-grade gliomas, but its efficacy is often limited by development of resistance^3^. Therefore, new medicines are needed for glioma treatment.

Autophagy that is characterized morphologically with formation of autophagosomes or autolysosomes in cytoplasm is a degradation pathway by which intracellular materials or impaired organelles are delivered to lysosomes for clearance^4^. Autophagy plays dual roles in regulation of cell destiny. On the one hand, it protects cells against detrimental stresses. On the other hand, it contributes to cell death, which is designated as autophagic death^4^. It was found that autophagy protected neuroblastoma cells by removing misfolded proteins, which were generated during the process of oxidative stress^5^. Autophagy was also reported to abrogate cisplatin-induced death in hepatocellular carcinoma cells via inhibiting accumulation of reactive oxygen species (ROS) by clearing damaged mitochondria^6^. Thus, the protection of autophagy against cell damage is closely associated with suppression of oxidative stress. In the case of autophagic death, autophagy is also accompanied with accumulation of intracellular ROS and generally thought to be activated by ROS^7,8^. However, it remains elusive whether autophagy could promote cell death via improving intracellular ROS.

Mitophagy refers to selective removal of damaged mitochondria via autophagy machinery^9^. Although mitophagy rescues cell death via clearing injured mitochondria^6^, accumulating evidence showed that excessive or sustained mitophagy could also lead to cell death^10,11^. It was reported that ceramide triggered glioma cell death via overactivation of mitophagy^10^. Moreover, mitophagy was found to contribute to the glioma cell death induced by chemical compound AT101^11^. Given that only damaged mitochondria could be recognized and degraded via autophagy pathway^9^, it remains elusive why excessive clearance of damaged mitochondria by autopahgy results in cell death. This makes us speculate that overactivated autophagy might play a role in causing mitochondrial damage.

**Fig. 1:**
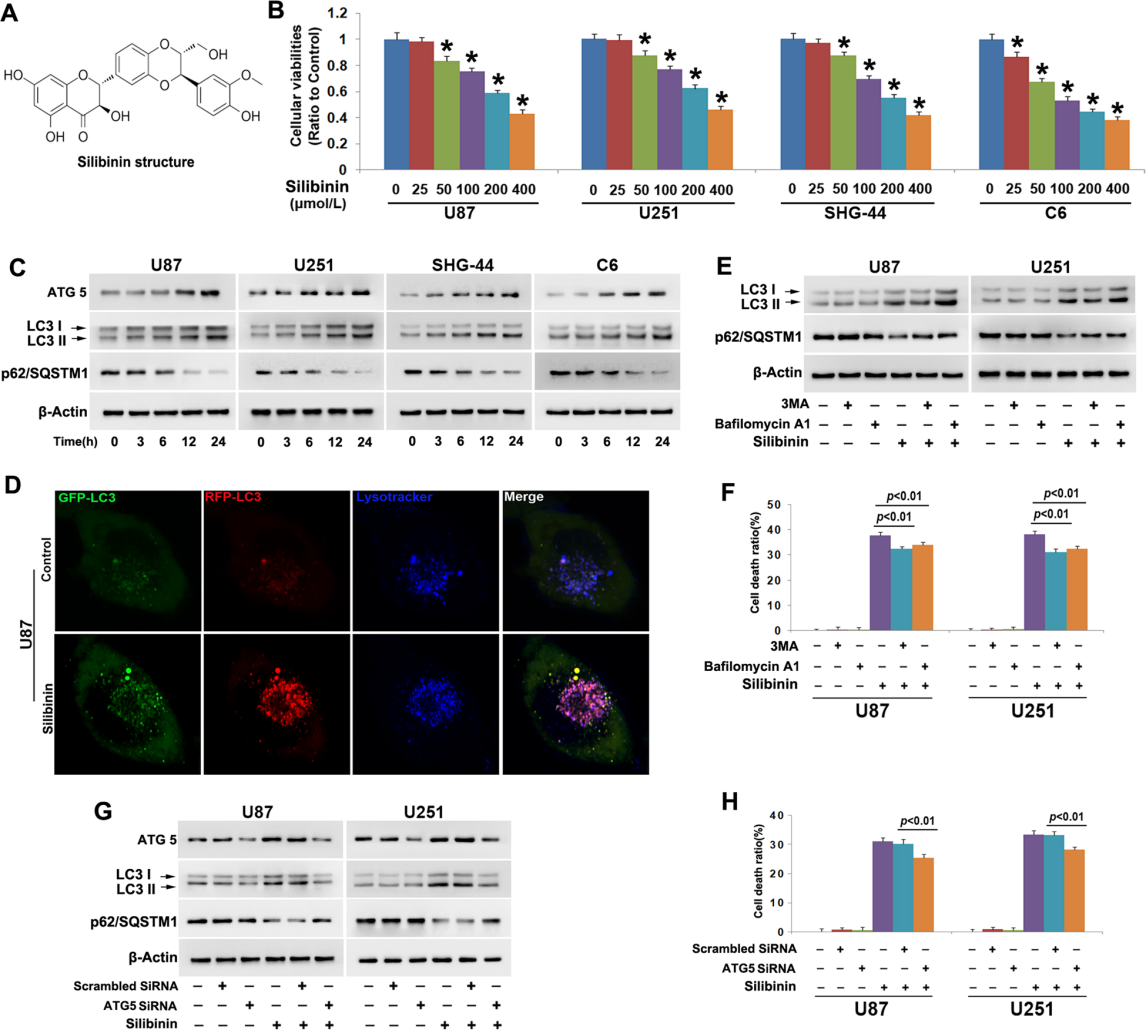
Silibinin induced autophagic death in glioma cells. **a** Silibinin structure. **b** MTT assay showed that silibinin inhibited the viabilities of U87, U251, SHG-44, and C6 glioma cells in a dosage-dependent manner. **c** Western blotting analysis showed that silibinin induced time-dependent upregulation of ATG5 and LC3-II, but downregulation of autophagy substrate p62(SQSTM1). **d** Representative images of the cells tranfected with StubRFP-SensGFP-LC3B lentivirus under confocal microscope revealed that silibinin induced formation of autophagosomes (yellow punta) and autolysosomes (violet puncta). **e** Western blotting analysis showed that 3MA abrogated silibinin-induced upregulation of LC3-II and reduction of p62. Bafilomycin A1 reversed silibinin-induced p62 reduction, but it further improved LC3-II levels. **f** LDH release assay proved that silibinin-induced glioma cell death was prevented in the presence of 3MA or bafilomycin A1. **g** Western blotting demonstrated that knockdown of ATG5 with SiRNA inhibited silibinin-induced upregulation of LC3-II and reduction of p62. **h** LDH release assay showed that silibinin-induced glioma cell death was abrogated when ATG5 was knocked down with SiRNA. The values are expressed as mean ± SEM (*n* = 5 per group).

Silibinin is a biologically active component of silymarin that is a polyphenolic extract from milk thistle (Silybum marianum) (Fig. 1a). It has been widely used to treat and prevent many types of hepatobiliary disorders including alcoholic liver disease, nonalcoholic fatty liver disease, and mushroom poisoning^12,13^. Moreover, silibinin not only exert potent inhibitory effect on various types of cancer cells, such as breast cancer cells, prostate cancer cells, and glioma cells^7,14,15^, but also sensitizes glioma cells to TMZ, TRAIL or arsenic trioxide treatment and prevents hepatocyte injury induced by chemotherapy agent cisplatin^16–19^. Although silibinin could induce autophagic death in cancer cells^7,8^, the biochemical events downstream silibinin-induced autophagy remains unclear. Therefore, in this study, we used glioma cell lines and nude mice with xenografted glioma to investigate whether autophagy plays a role in promoting mitochondria damage and its potential mechanism.

## Materials and methods

### Reagents

Silibinin was purchased from MedChemExpress (Monmouth Junction, NJ). Glutathione (GSH) was from Sigma (St.Louis, MO). Silibinin was dissolved in DMSO to a storage concentration of 200 mmol/L. GSK-872 was from Calbiochem company (Billerica, MA, USA). GKT137831, necrostatin-1(Nec-1), NSA (Necrosulfonamide), and ferrostatin-1(Fer-1) were from selleckchem Company (Houston, TX). Deferoxamine was from Abcam company (Cambridge, MA). Anti-AIF (#4642) and phospho-p53(Ser15) (#9284) antibody were from cell signaling company (Danvers, MA). The primary antibodies against the following proteins ATG5(ab109490), LC3B(192890) p62/SQSTM1(ab56416), xCT(ab175186), BNIP3(ab10433), PKM2(ab150377), HK II(ab209847), PFKP(ab119796), HIF-1α (ab51608), GPX4(ab125066), p53(ab26), TOMM20(ab186735), H2A(ab177308), and β-Actin (ab8226) were all purchased from Abcam company (Cambridge, MA). Other reagents were purchased from Sigma (St. Louis, MO).

### Cell line and culture

Human U87, U251, SHG-44, and rat C6 glioma cells were all obtained from Shanghai Institute of Cell Biology, Chinese Academy of Sciences (Shanghai, China). The cells were cultured in DMEM supplemented with 10% fetal bovine serum, 2 mmol/L glutamine, penicillin (100 U/mL) and streptomycin (100 μg/mL), and maintained at 37 °C and 5% CO_2_ in a humid environment. Cells in the mid-log phase were used in the experiments.

### Cellular viability and cell death assays

U87 (5 × 10^4^ cells/well), U251 (5 × 10^4^ cells/well), SHG-44 (5 × 10^4^ cells/well), and C6 (1 × 10^5^ cells/well) glioma cells were treated with target compounds after being seeded onto 96-well microplate with six duplicated wells and cultured 24 h. Cellular viability was assessed using an MTT assay, and was expressed as a ratio to the absorbance value at 570 nm of the control cells. Cell death was assessed by using lactate dehydrogenase cytotoxicity assay kit according to the manufacturer’s instructions (Beyotime Biotech, Nanjing, China), the absorbance value of each sample was read at 490 nm, and cell death ratio was calculated by using the following formula: cell death ratio % = (A sample − A control/A max − A control) × 100. A sample: sample absorbance value; A control: the absorbance value of control group; A max: the absorbance value of positive group.

### Measurement of mitochondrial membrane potential and mitochondrial superoxide

Mitochondrial membrane potential was assayed by using JC-1 staining (Beyotime Biotech, Nanjing, China) as described by manufacture’s instruction. The collected cells were analyzed by flow cytometry (FACScan, Becton Dickinson, San Jose, CA). Mitochondrial superoxide was assayed by using MitoSOX red according to manufacturer’s description (Invitrogen, Eugene, OR). The red fluorescence density was measured at an excitation wavelength of 510 nm and an emission wavelength at 580 nm, and was expressed as a ratio to the fluorescence in control cells. The cells seeded on a six-well plate were treated as the same as above described, and then observed under fluorescence microscope (Olympus IX71, Tokyo, Japan). All the measurements and observation were performed by a researcher who was blinded to group allocation and repeated for five times.

### Measurement of intracellular H_2_O_2_ content

The content of intracellular H_2_O_2_ in treated glioma cells was analyzed with a H_2_O_2_ assay kit (Beyotime Institute of Biotechnology, Nanjing, China) according to the manufacturer protocol. In brief, the cells were collected by centrifugation at 800 × *g* for 5 min and washed twice with PBS. The cell pellets and 10 mg xenograft glioma tissues were lysed in lysis buffer by repeated cycles of freezing and thawing under liquid nitrogen and centrifuged at 12,000 × *g* for 5 min. Then, 50 mL of supernatants and 100 mL of test solution were added into a tube, placed at room temperature for 30 min, and measured immediately with a spectrophotometer at a wavelength of 560 nm. Absorbance values were calibrated to a standard concentration curve to calculate the concentration of H_2_O_2_. The measurement was performed by a researcher who was blinded to group allocation and repeated for five times. Finally, the results were expressed as a ratio to the concentration of the control cells.

### Transfection of small interfering RNA (SiRNA)

The cells were seeded onto a culture dish. Transfection of siRNA was performed by using Lipofectamine 2000 (Invitrogen, Eugene, OR) according to the manufacturer’s instructions.

ATG5 SiRNA (5′-GACGUUGGUAACUGACAAATT-3′),

BNIP3 SiRNA (5′- GAUUACUUCUGAGCUUGCATT-3′),

AIF SiRNA (5′-GCAGUGGCAAGUUACUUAUTT-3′)

and scrambled SiRNA (5′-UUCUCCGAACGUGUCACGUTT-3′) were all purchased from GenePharma Company (Suzhou, China). After SiRNA transfection overnight, the cells were incubated with silibinin at indicated dosage for subsequent experiments.

### Rat C6 tumor xenograft in mice

The female athymic BALB/c nude mice (age 4 weeks, weight 20–22 g, Beijing Vital River laboratory animal technology company, China) were housed in a specific pathogen-free environment under the condition of 12-h light/12-h dark cycle, free access to food and water, and acclimatized to their surroundings for 3 days. The mice were cared in accordance with the guidelines for experimental animals of Jilin University and the study was approved by the ethics committee of First Hospital of Jilin University (Changchun, China). The animal quantity used in this study was estimated as described previously^20^. A total of 1 × 10^7^ logarithmically growing C6 cells in 100 μL of PBS were subcutaneously injected into the right flank of each mouse. After 7 days, the mice with similar tumor size (about 150 mm^3^) were randomly allocated to control group (*n* = 5) and treatment group (*n* = 5). The treatment group received intraperitoneal injections of silibinin at the dosage of 100 mg/kg body weight each day for 12 days, and the control group received vehicle in the same volume. The tumor size was measured by a researcher who was blinded to the group allocation with a slide caliper, and the tumor volume was calculated using the formula: 0.5 × A × B^2^, in which A is the length of the tumor and B is the width. On the next day of the last treatment, the mice were euthanized by cervical dislocation. After being excised and weighed, the tumors were frozen immediately in liquid nitrogen for western blotting analysis.

### Differential centrifugation, gel electrophoresis, and western blotting

The collected glioma cells by centrifugation and the frozen xenografted glioma tissue were homogenized with a glass Pyrex microhomogenizer (20 strokes) in ice cold lysis buffer (Beyotime Biotech, Nanjing, China). Homogenates were centrifuged at 1000 × *g* for 10 min at 4 °C to obtain the supernatant 1 and the pellet 2. The supernatant 1 was then centrifuged at 10,000 × *g* for 10 min at 4 °C to obtain supernatant 2 and pellet 2. The pellet 1 was nuclear fraction, supernatant 1 was cytoplasmic fraction, pellet 2 was mitochondrial fraction, and supernatant 2 was cytoplasmic fraction without mitochondria. The protein content was determined using Bio-Rad protein assay kit. After SDS electrophoresis and transfer to PVDF membranes, the membranes were blocked with 3% BSA in TBS for 30 min at room temperature, and then incubated overnight at 4 °C with primary antibodies. After incubation with horseradish peroxidase-conjugated secondary antibody and washing the blots, immunoreactive proteins were visualized on a chemi-luminescence developer (ChemiScope 5300, Clinx Scicence Instrument Company, Shanghai) and then the density was quantified by using software of Image J. The procedure was performed by a researcher who was blinded to group allocation.

### Immunocytochemical staining

The cells seeded on a culture dish were fixed in ethanol, washed with PBS, and incubated with 1%Triton X-100 for 10 min. The cells were incubated with 100 nmol/L Mitotracker red ((Invitrogen company, Eugene, OR)) for 30 min at 37 °C before fixation in ethanol. After the nonspecific antibody binding sites were blocked, the cells were incubated with anti-BNIP3 (1:100) or anti-AIF antibody (1:100) followed by incubation in Alexa Fluor 488-conjugated goat anti-rabbit IgG (1:200) for 1 h and then with Heochst33258. Finally, all the cells were visualized under laser scanning confocal microscope (Olympus FV1000, Tokyo, Japan) by a researcher who was blinded to group allocation.

### Measurement of GSH, cysteine, ATP, glucose-6-phophate, and pyruvate

Intracellular total GSH was measured by using a DTNB-GSSH reductase recycling assay kit (Beyotime Biotechnology, Nanjing, China) as described by manufacture. Briefly, the collected cells and frozen glioma tissue were resuspended in protein-removing buffer S and lysed by repeated cycles of freezing and thawing under liquid nitrogen. The cell lysates were centrifuged at 10,000 × *g* for 10 min at 4 °C to get the supernatant used for assay. GSH content was expressed as a ratio to the absorbance value at 412 nm of the control cells.

Intracellular cysteine was measured by using a cysteine assay kit (Nanjing Jiancheng Bioengineering Institute, Nanjing, China) according to the manufacturer protocol. Briefly, the collected cells were added into reagent A, homogenized on ice, and centrifuged at 8000 × *g* for 4 min at 4 °C to obtain the supernatant for assay. After the protein concentration was measured, 20 μL sample was incubated with 100 μL reagent B and 100 μL reagent C for 15 min at room temperature and read at absorbance 600 nm in a microplate reader. Finally, the results were expressed as a ratio to the absorbance value of the control cells.

Glucose-6-phosphate, pyruvate, and ATP measurement were all performed according to manufacturer instructions of Biovision company (Milpitas, CA). Briefly, the cells were collected by centrifugation or 10 mg xenograft glioma tissues were lysed in assay buffer by repeated sonification to be prepared to samples for examinations. For glucose-6-phosphate assay, the samples were mixed with respective reaction buffers and readings were taken at 450 nm. For measurement of the concentration of pyruvate and ATP, the samples were mixed with respective reaction buffers and read at absorbance 570 nm in a microplate reader. All the measurement was performed by researchers who were blinded to group allocation and repeated for five times. Finally, the results were expressed as a ratio to the absorbance value of the control cells.

### StubRFP-SensGFP-LC3 assay

The StubRFP-SensGFP-LC3 assay was performed according to manufacturer’s instruction (Genechem, Shanghai China). Briefly, the U87 cells (5 × 10^4^ cells/mL) seeded on a culture dish were incubated with the StubRFP-SensGFP-LC3B lentivirus (MOI = 5 × 10^6^ TU/mL). New medium was changed after 12 h of incubation. At transinfection 72 h, the cells were treated with silibinin, and then incubated with lysotracker blue or mitotracker deep red (Invitrogen company, Eugene, OR). Finally, the cells were observed by a researcher who was blinded to group allocation with a confocal laser scanning microscope (Olympus, Tokyo, Japan).

### Statistical analysis

All data represent at least four independent experiments and are expressed as mean ± SD. Statistical comparisons were made using one-way ANOVA. *P* values of less than 0.05 were considered to represent statistical significance.

## Results

### Autophagy contributed to silibinin-induced glioma cell death

To evaluate the toxic effect of silibinin on glioma cells, we used MTT assay to assess cellular viability. As shown in Fig. 1b, treatment with silibinin at indicated concentrations for 24 h resulted in obvious decreases in the cellular viabilities of U87, U251 and SHG-44 and C6 glioma cells, and the reduction of cellular viability became more apparent with the increase of silibinin concentration. This indicated that silibinin inhibited glioma cell viability in a dosage-dependent manner. Then, we calculated the IC50 values of silibinin at 24 h and found that they were 272.1 μmol/L in U87 cells, 261.6 μmol/L in U251 cells, 246.1 μmol/L in SHG-44 cells, and 162.5 μmol/L in C6 cells. Thus, 200 μmol/L was used in the subsequent studies.

To address the mechanism accounting for the inhibitory effect of silibinin on glioma cells, we investigated whether silibinin induced activation of lethal autophagy. As revealed by western blotting analysis, silibinin triggered time-dependent increases of autophagy marker proteins ATG5 and LC3-II, and corresponding reduction of autophagy substrate p62 (SQSTM1) (Fig. 1c and Fig. S1A–C). Then, the U87 cells transinfected with stubRFP-sensGFP-LC3 lentiviruses were used to evaluate whether autophagy flux was activated by silibinin in glioma cells. As revealed by confocal microscopy combined with lysosome probe lysotracker blue staining, many red pucta and green punta were found to form in the cytoplasm of the cells treated with silibinin at 200 μmol/L for 10 h, when compared with control cells (Fig. 1d). Although some red puncta were colocalized with green puncta to display yellow color, the other red puncta were colocalized with blue puncta to present violet color (Fig. 1d). Because green fluorescence is prone to be quenched in the acid environment of lysosome lumen, the yellow puncta were autophagosomes and the violet puncta were autolysosomes. Therefore, these results indicated that silibinin activated autophagy.

To assay the role of autophagy in silibinin-induced glioma cell death, U87 and U251 cells were treated for 1 h with 3MA that inhibits autophagy at initiation stage or bafilomycin A1 that disturbs autophagosome to fuse with lysosome, and then incubated with silibinin at 200 μmol/L for 24 h. Western blotting analysis showed that silibinin-induced upregulation of LC3-II and downregulation of p62 (SQSTM1) were both obviously inhibited by pretreatment with 5 mmol/L 3MA. Despite the reduction of p62 (SQSTM1) induced by silibinin was markedly suppressed in the cells pretreated with 1.5 μmol/L bafilomycin A1, the protein level of LC3-II was further improved (Fig. 1e, Fig. S1D and E). Then, LDH release assay demonstrated that silibinin-induced death in glioma cells was significantly abrogated in the presence of 3MA or bafilomycin A1 (Fig. 1f). To further verify the role of autophagy in silibinin-induced glioma cell death, we knocked down ATG5 with SiRNA and examined its effect on glioma cell death. It was found that knockdown of ATG5 not only prevented silibinin-induced upregulation of LC3-II and downregulation of p62 (SQSTM1) (Fig. 1g, Fig. S1F–H), but also inhibited glioma cell death (Fig. 1h). Therefore, these results indicated that silibinin induced autophagic death in glioma cells.

Furthermore, we used SHG-44 cells to investigate whether silibinin induced necroptosis and ferroptosis in glioma cells. As shown by LDH assay, 1 h pretreatment with necroptosis specific inhibitor Nec-1 (100 μmol/L), GSK-872 (20 μmol/L), and NSA (10 μmol/L) did not prevent silibinin-induced glioma cell death (Fig. S1I). Additionally, pretreatment with ferroptosis inhibitor deferoxamine (500 μmol/L) and Fer-1 (20 μmol/L) did not inhibit the glioma cell death induced by silibinin as well (Fig. S1J). Thus, these results indicated that neither necroptosis nor ferroptosis was induced in glioma cells by silibinin.

### Autophagy contributed to silibinin-induced mitochondria damage

**Fig. 2:**
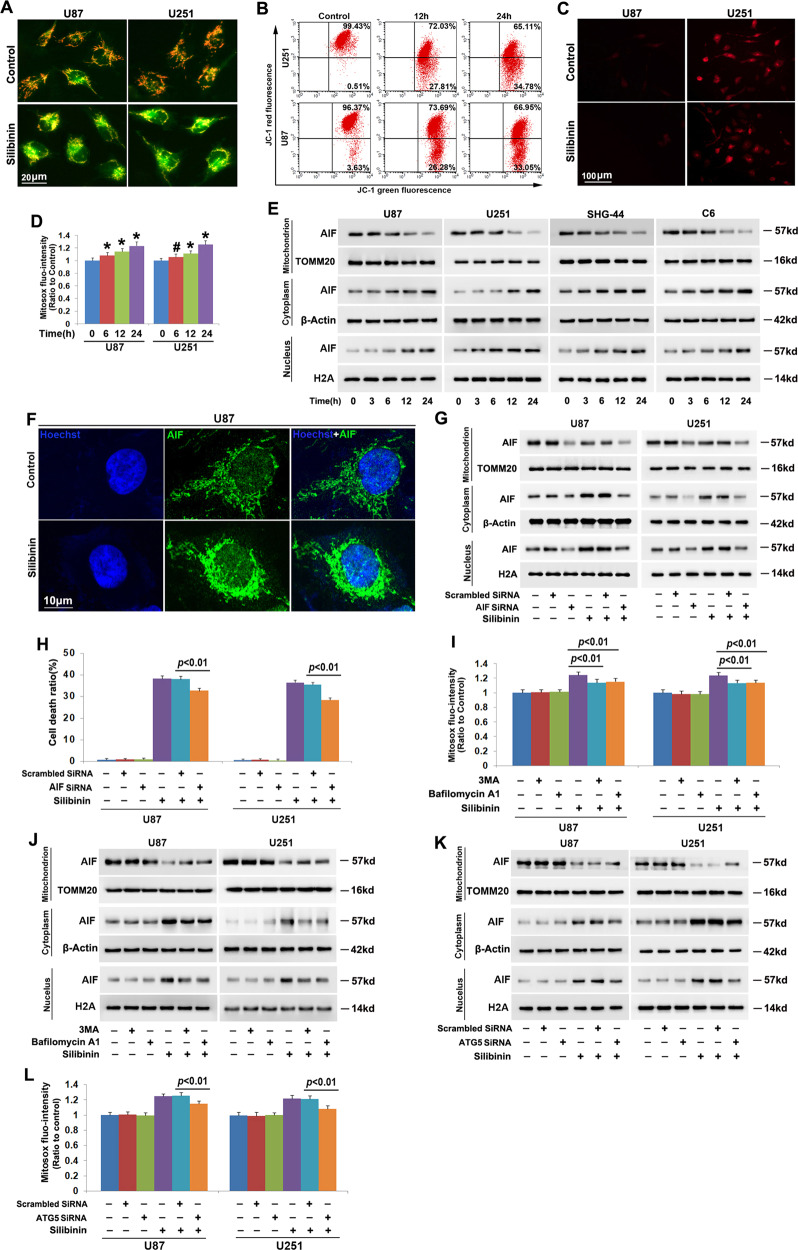
Autophagy contributed to silibinin-induced mitochondria damage. **a** Representative images of the cells stained with JC-1 under fluorescence microscope showed that silibinin treatment resulted in obvious reduction of red fluorescence in U87 and U251 cells. **b** Flow cytometry analysis combined with JC-1 staining confirmed that silibinin induced time-dependent dissipation of mitochondria membrane potentials. **c** Representative images of the cells stained with Mitosox red under fluorescence microscope showed that the red fluorescence exhibited by Mitosox red was apparently stronger in silibinin-treated cells than that in control group. **d** Statistical analysis of the red fluorescence intensity exhibited by Mitosox red proved that silibinin triggered mitochondrial accumulation of superoxide in a time-dependent manner. **e** Western blotting analysis revealed that silibinin induced AIF translocation from mitochondria to nuclei in a time-dependent manner. **f** Representative images acquired by confocal microscopy combined with immunochemical staining showed that silibinin induced accumulation of AIF in the nucleus of U87 cell. **g** Silibinin-induced accumulation of AIF in nuclear fraction was decreased in the cells transfected with AIF SiRNA. **h** LDH release assay proved that knockdown of AIF prevented silibinin-induced glioma cell death. **i** Statistical analysis of the red fluorescence intensity exhibited by Mitosox red showed that silibinin-induced mitochondrial accumulation of superoxide was significantly inhibited in the presence of 3MA or bafilomycin A1. **j** Western blotting proved that knockdown of ATG5 with SiRNA prevented silibinin-induced AIF translocation from mitochondria to nuclei. **k** Western blotting revealed that silibinin-induced nuclear translocation of AIF was suppressed when ATG5 was knocked down with SiRNA. **l** Knockdown of ATG5 with SiRNA abrogated the improvement of mitochondrial superoxide induced by silibinin.

To address whether silibinin induces damage in mitochondrion, we measured mitochondrial membrane potential by using JC-1 and mitochondrial superoxide by using Mitosox red. JC-1is a probe emitting red fluorescence when accumulates in healthy mitochondria but green fluorescence when is released from damaged mitochondria into cytoplasm. As revealed by fluorescence microscopy, the red fluorescence exhibited by JC-1 decreased drastically in the cells treated with silibinin at 200 μmol/L for 12 h, when compared with control cells (Fig. 2a). This was also demonstrated by the results acquired by flow cytometry analysis combined with JC-1 staining, which showed that silibinin caused time-dependent reduction in red fluorescence (Fig. 2b, Fig. S2A). These indicated that silibinin induced mitochondria depolarization in a time-dependent manner. Then, we found that the red fluorescence exhibited by Mitosox red which is a specific probe for mitochondrial superoxide was much brighter in the cells treated with silibinin at 200 μmol/L for 24 h than that in control cells (Fig. 2c). Statistical analysis demonstrated as well that the red fluorescence intensity was improved with the extension of silibinin treatment (Fig. 2d). This indicated that silibinin induced time-dependent accumulation of mitochondrial superoxide. Therefore, these data suggested that silibinin treatment resulted in mitochondrial damage.

Considering that AIF translocation from depolarized mitochondria to nuclei could lead to cell death, we thus isolated mitochondrial and nuclear fractions and examined silibinin-induced changes in distribution of AIF. As revealed by western blotting, when compared with control cells, AIF was decreased in mitochondria fractions, whereas increased in nuclear fractions by silibinin in a time-dependent manner (Fig. 2e, Fig. S2B–D). Confocal microscopy showed as well that AIF accumulated more apparently in the nuclei of the cells incubated with silibinin for 24 h than that in control ones (Fig. 2f). To clarify the role of AIF in silibinin-induced glioma cell death, we introduce SiRNA to knock down AIF and examined its effect on glioma cell death by using LDH release assay. It was found that silibinin-induced upregulation of AIF in nuclear fractions was mitigated as a consequence of the overall reduction of AIF expression in the cells transfected with AIF SiRNA when compared with the cells transfected with scrambled SiRNA (Fig. 2g, Fig. S2E–G). Moreover, silibinin-induced glioma cell death was also suppressed significantly when AIF was knocked down with SiRNA (Fig. 2h). Therefore, these indicated that silibinin triggered AIF-dependent death in glioma cells.

Furthermore, we found that inhibition of autophagy with 3MA or bafilomycin A1 obviously abrogated silibinin-induced accumulation of mitochondrial superoxide and AIF translocation from mitochondria to nuclei (Fig. 2i and j, Fig. S2H–J). Similar results could be found when ATG5 was knocked down with SiRNA (Fig. 2k and l, Fig. S2K–M). Therefore, these results suggested that autophagy contributed to silibinin-induced mitochondria damage and nuclear translocation of AIF.

### Autophagy reinforced silibinin-induced BNIP3 accumulation on mitochondria

**Fig. 3:**
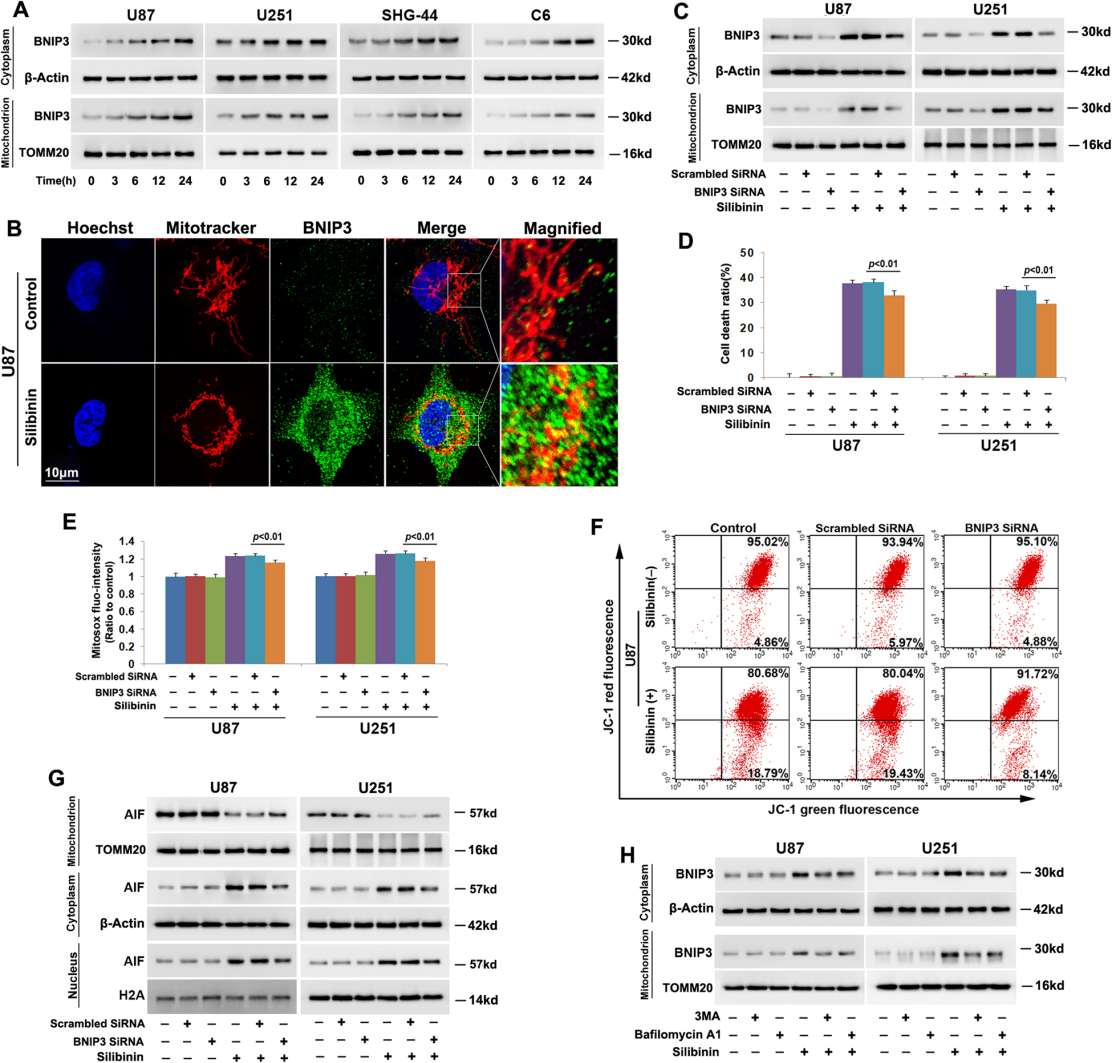
Autophagy promoted silibinin-induced BNIP3 expression and accumulation on mitochondria. **a** Western blotting analysis showed that silibinin induced BNIP3 upregulation and promoted BNIP3 accumulation on mitochondria in a time-dependent manner. **b** Representative images acquired by confocal microscopy combined with immunocytochemical staining showed that the upregulated BNIP3 induced by silibinin were colocalized with mitochondria. **c** Silibinin-induced BNIP3 upregulation and its accumulation on mitochondria were both inhibited in the cells transfected with BNIP3 SiRNA. **d** LDH release assay showed that silibinin-induced glioma cell death was prevented when BNIP3 was knocked down with SiRNA. **e** Statistical analysis of the intensity of the red fluorescence exhibited by the cells incubated with Mitosox red proved that knockdown of BNIP3 with SiRNA prevented silibinin-induced accumulation of mitochondrial superoxide. **f** Flow cytometry analysis combined with JC-1 staining showed that knockdown of BNIP3 alleviated silibinin-induced mitochondria depolarization. **g** Western blotting analysis revealed that silibinin-induced AIF translocation from mitochondria to nuclei was suppressed when BNIP3 was knocked down with SiRNA. **h** Western blotting proved that silibinin-induced BNIP3 upregulation and accumulation on mitochondria were both inhibited in the presence of 3MA or bafilomycin A1. *p < 0.01 versus control group. The values are expressed as mean ± SEM (*n* = 5 per group).

Given that BNIP3 (Bcl-2/adenovirus E1B 19-kDa-interacting protein 3) is protein targeting mitochondria and could induce mitochondrial damage and nuclear translocation of AIF^6^, we thus isolated mitochondrial fractions from U87, U251, SHG-44, and C6 cells and examined silibinin-induced changes in BNIP3. As shown by western blotting, silibinin not only induced BNIP3 overexpression, but also improved BNIP3 levels in mitochondrial fractions in a time-dependent manner (Fig. 3a, Fig. S3A and B). Confocal microscopy also confirmed that BNIP3 accumulated more obviously on mitochondria in the cells treated with silibinin for 24 h than that in control cells (Fig. 3b). These indicated that silibinin promoted BNIP3 accumulation on mitochondria, as well as upregulated its expression in glioma cells. To elucidate the role of BNIP3 in silibinin-produced toxicity in glioma cells, we used SiRNA to knock down BNIP3 and examined its effect on glioma cell death and mitochondrial damage. As revealed by western blotting, silibinin-induced BNIP3 overexpression and its accumulation on mitochondria were both inhibited in the cells transfected with BNIP3 SiRNA, when compared with the cells transfected with scrambled SiRNA (Fig. 3c, Fig. S3C and D). Moreover, LDH release assay proved that knockdown of BNIP3 with SiRNA significantly prevented the glioma cell death induced by silibinin (Fig. 3d). Furthermore, silibinin-induced accumulation of mitochondrial superoxide, mitochondria depolarization, and AIF translocation from mitochondria to nuclei were all inhibited when BNIP3 was knocked down with SiRNA (Fig. 3e–g, Fig. S3E–G). Thus, these indicated that BNIP3 contributed to silibinin-induced glioma cell death via causing mitochondrial damage and nuclear translocation of AIF.

Notably, we found that silibinin-induced overexpression of BNIP3 and BNIP3 upregulation in mitochondrial fractions were both inhibited in the cells pretreated with 3MA or bafilomycin A1 (Fig. 3h, Fig. S3H and I). This indicated that autophagy promoted silibinin-induced BNIP3 overexpression and its accumulation on mitochondria. This might be a factor accounting for the promotion effect of autophagy on silibinin-induced mitochondria.

### Autophagy augmented silibinin-induced accumulation of H2O2

**Fig. 4:**
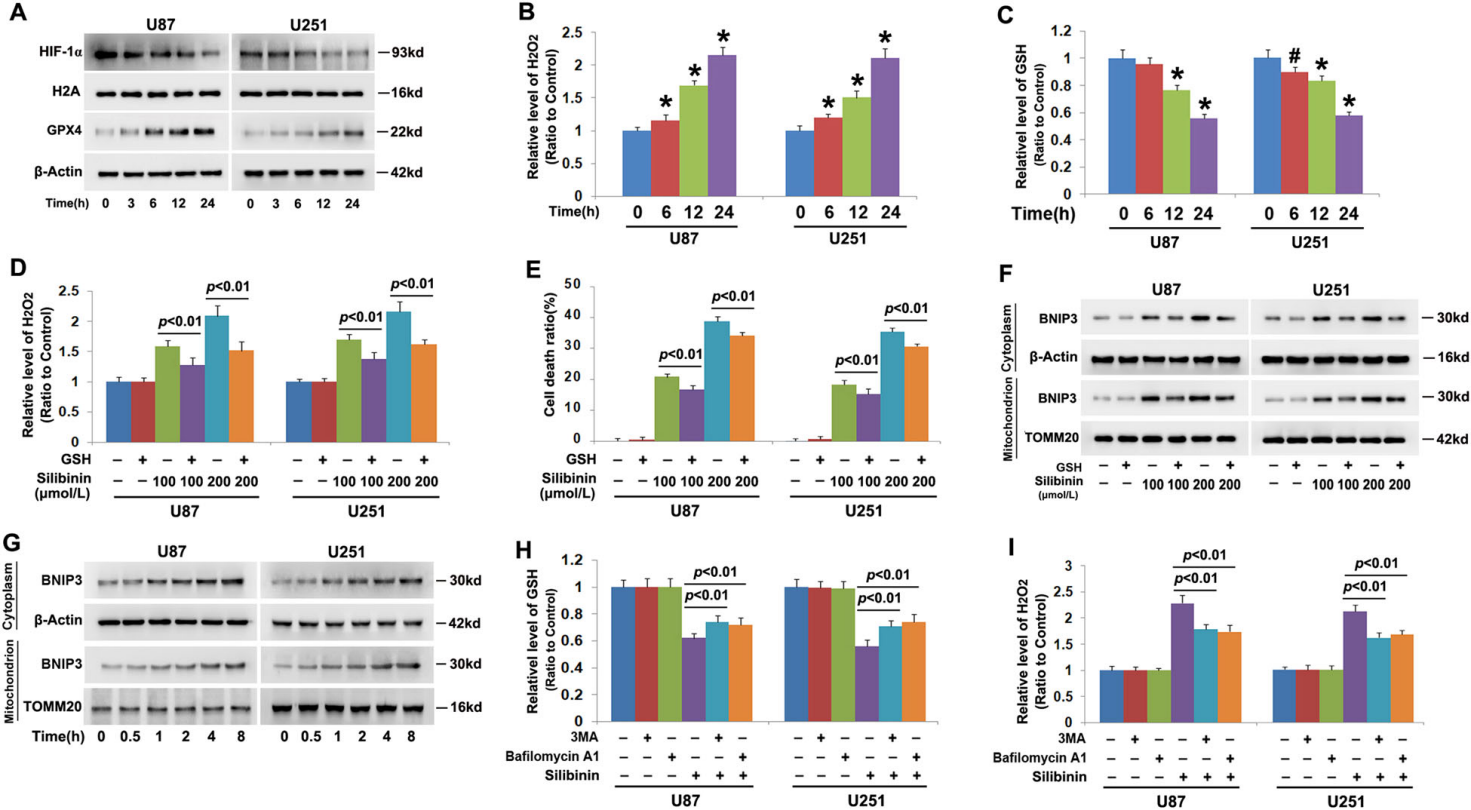
Autophagy promoted silibinin-induced increase of H_2_O_2_ via causing GSH depletion. **a** Western blotting proved that silibinin induced downregulation of HIF-1α, but upregulation of GPX4 in a time-dependent manner. **B** H_2_O_2_ assay showed that intracellular H_2_O_2_ was improved time-dependently in silibinin-treated glioma cells. **c** GSH assay proved that silibinin treatment resulted in time-dependent depletion of GSH. **d** H_2_O_2_ assay demonstrated that supplement of exogenous GSH prevented silibinin-induced increase of H_2_O_2_. **e** LDH release assay showed that silibinin-induced glioma cell death was prevented when GSH was supplemented. **f** Western blotting revealed that pretreatment with GSH prevented silibinin-induced BNIP3 overexpression and accumulation on mitochondria. **g** Western blotting showed that H_2_O_2_ not only induced time-dependent upregulation of BNIP3, but also improved BNIP3 levels in mitochondrial fractions in a time-dependent manner. **h** GSH assay showed that silibinin-induced GSH depletion was inhibited in the presence of 3MA or bafilomycin A1. **i** H_2_O_2_ assay showed silibinin-induced improvement of H_2_O_2_ was reversed in the cells pretreated with 3MA or bafilomycin A1. ^#^*p* < 0.05 versus control group; *p < 0.01 versus control group. The values are expressed as mean ± SEM (*n* = 5 per group).

To address the mechanism accounting for the regulatory effect of silibinin on BNIP3, we examined silibinin-induced changes in HIF-1α and hydrogen peroxide given that HIF-1α is a transcription factor of BNIP3 and BNIP3 could also be upregulated under the condition of oxidative stress^6^. Thus, we isolated nuclear fractions by using differential centrifugation and examined the protein level of HIF-1α in the absence and presence of silibinin by western blotting. When compared that in control cells, nuclear level of HIF-1α was downregulated by silibinin in a time-dependent manner (Fig. 4a, Fig. S4A). However, intracellular hydrogen peroxide was markedly improved time-dependently by silibinin, which was accompanied with GSH depletion at each indicated time point (Fig. 4b). Considering that NADPH oxidase (Nox) plays a role in promoting the generation of hydrogen peroxide, the cells were pretreated with Nox inhibitor GKT137831 at 500 μmol/L for 1 h and then incubated with silibinin for 24 h. It was found that GKT13831 not only inhibited silibinin-induced improvement of hydrogen peroxide, but also prevented glioma cell death (Fig. S4B and C). This indicated that Nox might be involved in regulation silibinin-induced overproduction of hydrogen peroxide. Furthermore, we found that silibinin treatment also resulted in time-dependent upregulation of glutathione peroxidase 4 (GPX4) (Fig. 4a, Fig. S4D). Given that GSH could be used by GPX4 to clear hydrogen peroxide^17^, the glioma cells were supplemented with exogenous GSH to verify the role of hydrogen peroxide in silibinin-induced changes in BNIP3. It was found that pretreatment with GSH at 10 mmol/L for 1 h obviously inhibited the improvement of hydrogen peroxide induced by silibinin at indicated dosages (Fig. 4d). Moreover, LDH release assay demonstrated as well that silibinin-induced glioma cell death was abrogated significantly by supplement of GSH (Fig. 4e). Then, it was revealed by western blotting that silibinin-induced overexpression of BNIP3 in cytoplasmic and mitochondrial fractions were both inhibited in the presence of GSH (Fig. 4f, Fig. S4E and F). To further confirm the role of hydrogen peroxide in promoting BNIP3 expression and accumulation on mitochondria, we treated U87 and U251 cells with exogenous hydrogen peroxide at 300 μmol/L for indicated time and analyzed the distribution of BNIP3 by western blotting. It was found that hydrogen peroxide not only upregulated BNIP3 expression, but also improved BNIP3 levels in mitochondrial fractions in a time-dependent manner (Fig. 4g, Fig. S4G and F). These results indicated that GSH depletion contributed to silibinin-induced improvement of hydrogen peroxide, which resulted in BNIP3 upregulation and accumulation on mitochondria.

Additionally, we found that pretreatment with 3MA or bafilomycin A1 not only reversed silibinin-induced GSH depletion, but also prevented accumulation of hydrogen peroxide (Fig. 4h and i). Therefore, these results suggested that autophagy contributed to silibinin-induced changes in BNIP3 via causing GSH depletion-dependent accumulation of hydrogen peroxide.

### Autophagy promoted silibinin-induced phosphorylation of p53

**Fig. 5:**
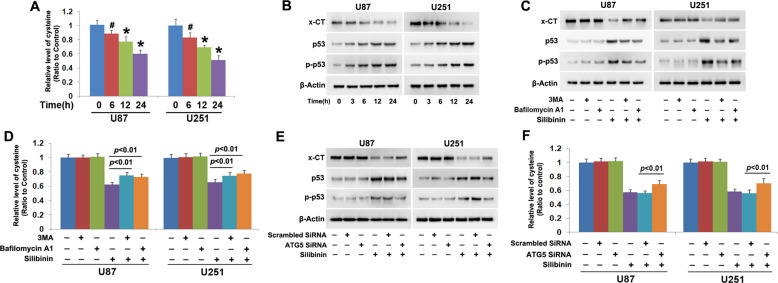
Autophagy promoted silibinin-induced cystine depletion and p53 phosphorylation. **a** Cysteine assay showed that silibinin induced time-dependent depletion of cysteine. **b,** **c** Western blotting analysis proved that silibinin downregulated xCT, but upregulated p53 and phospho-p53 in a time-dependent manner. These were all prevented in the presence of 3MA or bafilomycin A1. **d** Cysteine assay demonstrated that silibinin-induced depletion of cysteine was prevented in the presence of 3MA or bafilomycin A1. **e** Western blotting demonstrated that knockdown of ATG5 with SiRNA prevented silibinin-induced downregulation of CT and upregulation of p53 and phospho-p53. **f** Cysteine assay proved that silibinin-induced depletion of cysteine was inhibited when ATG5 was knocked down with SiRNA. ^#^*p* < 0.05 versus control group; *p < 0.01 versus control group. The values are expressed as mean ± SEM (*n* = 5 per group).

To elucidate why silibinin treatment leads to GSH depletion, we examined silibinin-induced changes in cysteine because it is a material used for GSH synthesis^21^. As shown in Fig. 5a, intracellular cysteine was decreased by silibinin in a time-dependent manner, when compared with that in control cells. Considering that cysteine is transformed from cystine whose transportation into cells depends on cystine/glutamate antiporter^21^, we used western blotting to analyze silibinin-induced changes in the protein level of xCT (SLC7A11) and phosphorylation of p53. xCT is a specific light-chain subunit of cystine/glutamate antiporter and its expression is negatively regulated by phosphorylated p53^21^. We found that silibinin downregulated xCT, but upregulated p53 and phospho-p53 in a time-dependent manner (Fig. 5b, Fig. S5A–C). These indicated that silibinin activated p53, which resulted in downregulation of xCT and depletion of cysteine.

In contrast, the downregulation of xCT and the phosphorylation of p53 induced by silibinin were both inhibited apparently when the cells were pretreated with 3MA or bafilomycin A1 (Fig. 5c, Fig. S5D–F). Moreover, silibinin-induced depletion of cysteine was correspondingly prevented in the presence of 3MA or bafilomycin A1 (Fig. 5d). Similar results could be found when ATG5 was knocked down with SiRNA (Fig. 5e and f). Thus, these data indicated that autophagy contributed to silibinin-induced depletion of cysteine via promotion of p53 activation.

### Silibinin suppressed glycolysis in glioma cells

**Fig. 6:**
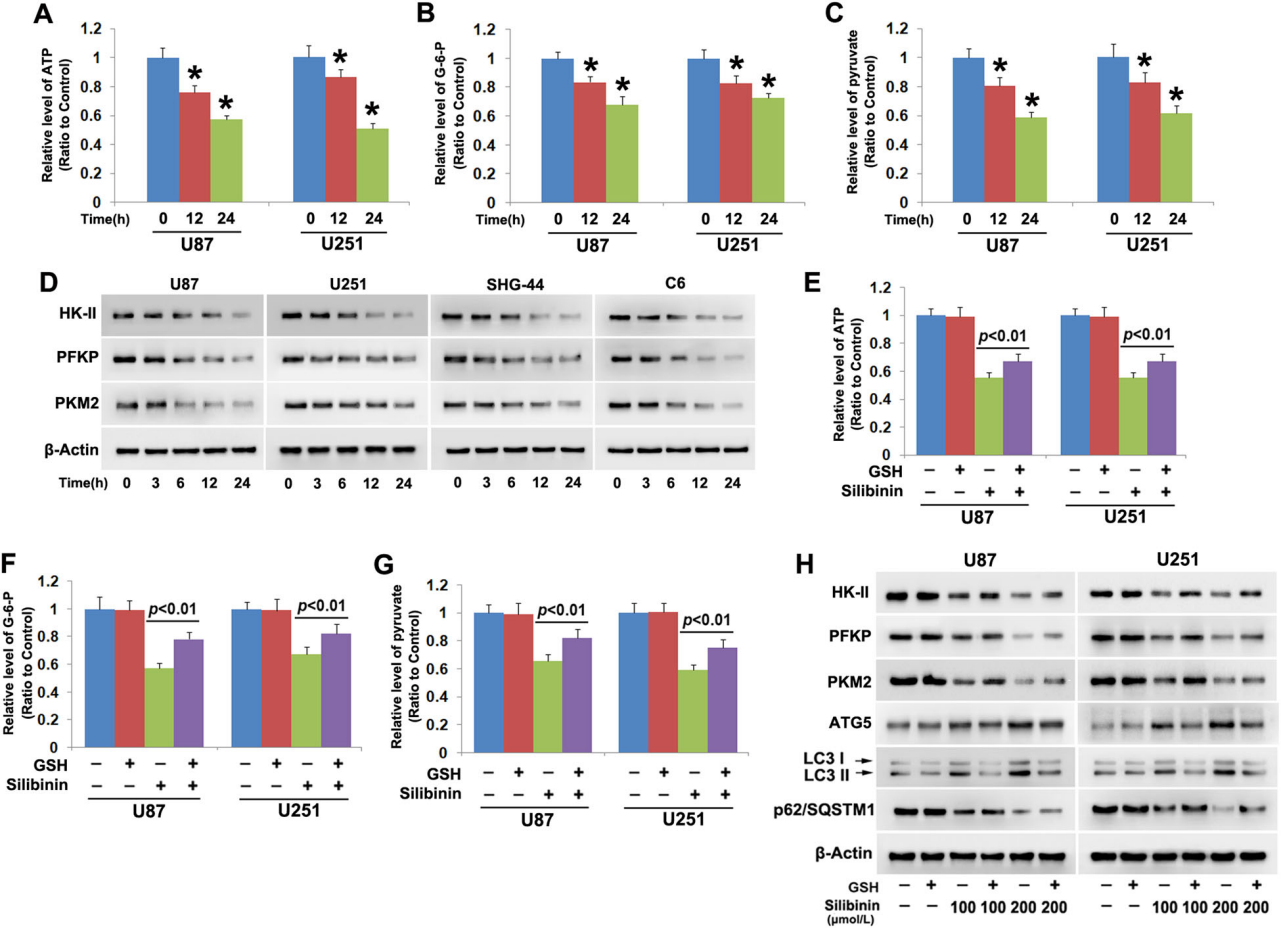
Silibinin suppressed glycolysis in glioma cells. **a**–**c** Silibinin depleted ATP, glucose-6-phosph and pyruvate in a time-dependent manner. **d** Western blotting revealed that silibinin induced time-dependent downregulation of HK II, PFKP, and PKM2. **e**–**g** Pretreatment with GSH prevented silibinin-induced depletion of ATP, glucose-6-phosph, and pyruvate. **h** Western blotting proved that the downregulation of HK II, PFKP, PKM2, and p62 and the upregulation of ATG5 and LC3-II induced by silibinin were all inhibited in the presence of GSH. *p < 0.01 versus control group. The values are expressed as mean ± SEM (*n* = 5 per group).

To elucidate the mechanism accounting for silibinin-induced autophagy activation, we investigated whether silibinin induces glycolysis dysfunction considering that glycolysis is the primary energy-generating pathway in cancer cells and energy failure could lead to autophagy activation^4^. It was found that silibinin triggered time-dependent depletion of ATP (Fig. 6a). Moreover, glycose-6-phosphate, which is the first product of glycolysis generated by hexokinase II (HK II) and pyruvate that is the final product of glycolysis produced by pyruvate kinase 2 (PKM2) were both depleted by silibinin in a time-dependent manner (Fig. 6b and c). These indicated that silibinin suppressed glycolysis in glioma cells. Then, western blotting was used to analyze silibinin-induced changes in HK II, 6-phosphofructokinase (PFKP), and PKM2, which are known to regulate the three rate-limiting steps of glycolysis. We found that the protein levels of HK II, PFKP, and PKM2 were all downregulated time-dependently by silibinin in U87, U251, SHG-44, and C6 glioma cells (Fig. 6d, Fig. S6A–C). This indicated that silibinin suppressed glycolysis via downregulation of HK II, PFKP, and PKM2.

To test the role of hydrogen peroxide in silibinin-induced glycolysis dysfunction, U87 and U251 cells were treated with antioxidant GSH at 10 mmol/L for 1 h and then incubated with silibinin at 200 μmol/L for 24 h. It was found that silibinin-induced reduction of ATP, glucose-6-phosphate, and pyruvate were all inhibited by supplement of GSH (Fig. 6e–g). Western blotting analysis revealed that pretreatment with GSH obviously abrogated the downregulation of HK II, PFKP, and PKM2 induced by silibinin at indicated dosages (Fig. 6h, Fig. S6D–F). Correspondingly, silibinin-induced increases of autophagy marker proteins ATG5 and LC3-II and decrease of autophagy substrate p62 (SQSTM1) were all inhibited in the presence of GSH (Fig. 6h, Fig. S6G–I). Thus, these data not only indicated that hydrogen peroxide reinforced the inhibitory effect of silibinin on glycolysis, but also indirectly suggested that glycolysis dysfunction led to silibinin-induced autophagy activation.

### Silibinin inhibited glioma cell growth in vivo

**Fig. 7:**
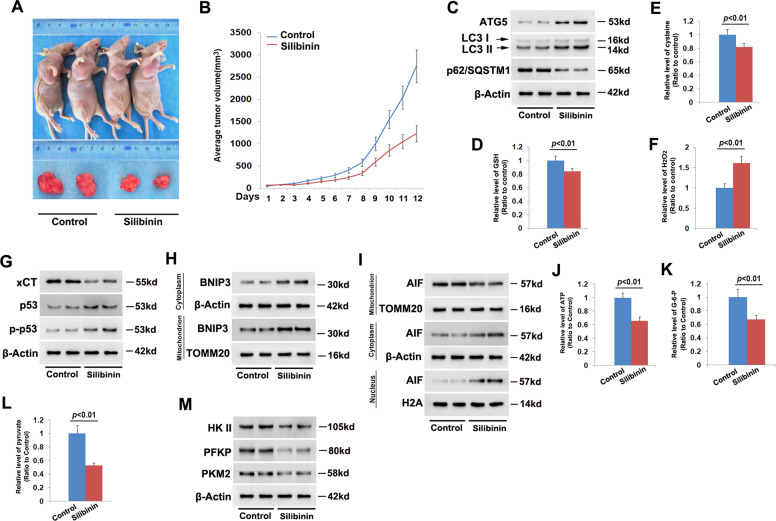
Silibinin inhibited glioma cell growth in vivo. **a** Representative images of the mice with xenografted gliomas and the removed tumors. **b** Statistical analysis of silibinin-induced changes in tumor volumes. The average tumor size was 2750 ± 406 mm^3^ in control group, which reduced to 1225 ± 157 mm^3^ after being treated with silibinin for 12 days (*n* = 5). **c** Western blotting analysis revealed that silibinin upregulated ATG5 and LC3-II, but downregulated p62. **d**–**f** Silibinin treatment resulted in depletion of GSH and cysteine, but improvement in H_2_O_2_. **g** Western blotting analysis showed that silibinin downregulated xCT, but upregulated p53 and phospho-p53. **h** Silibinin triggered BNIP3 upregulation and accumulation on mitochondria. **i** Silibinin promoted AIF translocation from mitochondria to nuclei. **j**–**l** Silibinin significantly induced depletion of ATP, glucose-6-phosph, and pyruvate. **m** Silibinin treatment resulted in downregulation of HK II, PFKP, and PKM2. The values are expressed as mean ± SEM (*n* = 5 per group).

To verify the treatment effect of silibinin on glioma cells in vivo, C6 cells were xenografted subcutaneously into the flank of nude mice. After being treated with silibinin at the dosage of 100 mg/kg each day for consecutive 12 days, the volumes of the xenografted tumors were found to decrease markedly when compared with those in control group (Fig. 7a). This was also confirmed by statistical analysis of tumor volumes (Fig. 7b). Then, the tumors removed from the sacrificed mice were homogenized and silibinin-induced changes in autophagy marker proteins were analyzed by western blotting. When compared with the control group, silibinin triggered obviously upregulation of ATG5 and LC3-II, but reduction of p62 (SQSTM1) (Fig. 7c, Fig. S7A). This indicated that silibinin induced autophagy activation in glioma cells in vivo.

Moreover, it was found that hydrogen peroxide was at higher level, whereas GSH and cysteine were both at lower levels in silibinin-treated group than control group (Fig. 7d–f). Consistently, western blotting analysis revealed that xCT was markedly downregulated, but p53 and phospho-p53 were both obviously upregulated by silibinin (Fig. 7g, Fig. S7B). This indicated silibinin-induced accumulation of hydrogen peroxide, depletion of GSH and cysteine, downregulation of xCT and activation of p53 in glioma cells in vivo.

Furthermore, we found that silibinin not only upregulated the expression of BNIP3, but also improved BNIP3 levels in mitochondrial fractions (Fig. 7h, Fig. S7C). Although AIF was decreased by silibinin in mitochondrial fractions, it was increased apparently in nuclear fractions (Fig. 7i, Fig. S7D). This indicated that BNIP3 might play a role in regulation of silibinin-induced nuclear translocation of AIF in glioma cells in vivo. Additionally, it was found that silibinin treatment resulted in depletion of ATP, glucose-6-phosphate, and pyruvate (Fig. 7j–l) and downregulation of HK II, PFKP, and PKM2 (Fig. 7m, Fig. S7E). This indicated that silibinin triggered energy failure via suppression of glycolysis in glioma cells in vivo.

## Discussion

**Fig. 8:**
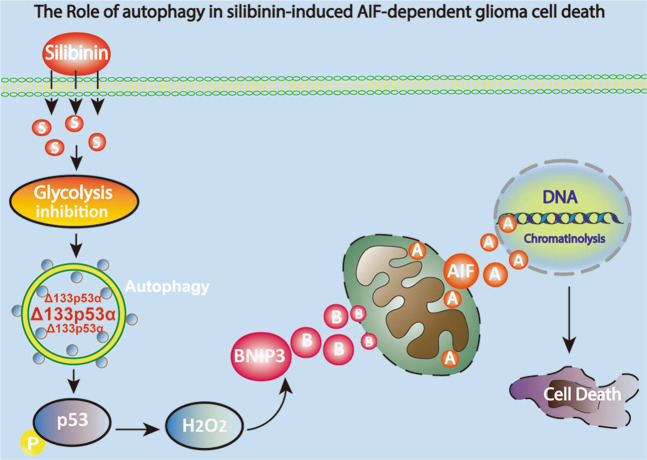
Schematic diagram for the role of autophagy in silibinin-induced mitochondrial damage and AIF-dependent glioma cell death. Silibinin inhibits glycolysis in glioma cells, which results in autophagy activation. The activated autophagy selectively degrades Δ133p53α (an inhibitoryp 53 isoform) to improve p53 phosphorylation. Then, the phosphorylated p53 reinforces generation of hydrogen peroxide, which further promotes the expression of BNIP3. BNIP3 damages mitochondria and causes AIF translocate from mitochondrion into nucleus. Finally, AIF leads to cell death via causing chromatinolysis.

In summary, we found in this study that silibinin induced AIF translocation from mitochondria to nuclei in glioma cells in vitro and in vivo, which was accompanied with autophagy activation. In vitro studies revealed that blocking autophagy with 3MA1, bafilomycin A1 or by knocking down ATG5 with SiRNA inhibited silibinin-induced mitochondrial accumulation of superoxide, AIF translocation from mitochondria to nuclei and glioma cell death. Mechanistically, silibinin activated autophagy through depleting ATP by suppressing glycolysis. Then, autophagy improved H_2_O_2_ via promoting p53-mediated depletion of GSH and cysteine and downregulation of xCT. The increased H_2_O_2_ not only contributed to silibinin-induced BNIP3 upregulation and translocation to mitochondria, but also exacerbated glycolysis dysfunction. Knockdown of BNIP3 with SiRNA inhibited silibinin-induced mitochondrial depolarization, accumulation of mitochondrial superoxide, and AIF translocation from mitochondria to nuclei, as well as prevented glioma cell death. Collectively, autophagy contributed to silibinin-induced glioma cell death via causing BNIP3-mediated mitochondria damage and nuclear translocation of AIF (Fig. 8).

Overactivation of autophagy has been established to be an effective way to eradicate cancer cells^7,8,22^, and silibinin is confirmed to be a potent lethal autophagy inducer for various types of cancer cells such as human breast cancer cells, fibrosarcoma cells, and renal carcinoma cells^7,8,23^. Different from previous report showing that silibinin triggered protective autophagy in glioma cells^24^, we found in this study that blocking autophagy with 3MA, bafilomycin A1 or by knocking down ATG5 with SiRNA significantly rescued silibinin-induced glioma cell death. This verified that autophagy contributed to silibinin-induced death in glioma cells. Moreover, different types of programmed cell death could be induced by the same chemical or under the same pathological condition. Previous reports have shown that silibinin treatment could also trigger apoptosis in glioma cells^15^. Additionally, accumulating evidences have demonstrated that autophagic death could occur simultaneously with other types of programmed cell death, such as ferroptosis and necroptosis^25,26^. Thus, induction of autophagic death is a pathway accounting for the toxic effect of silibinin on glioma cells.

Energy failure is a key factor initiating autophagy via activation of AMPK/mTOR pathway^4^. Glycolysis regulated by three rate-limiting enzymes HK II, PFKP, and PKM2 is usually used by cancer cells to acquire ATP^20^. Suppression of glycolysis by pharmacological inhibition of HK II and genetic knockdown of PKM2 were respectively reported to causing cancer cell death via excessive activation of autophagy^27,28^. In this study, we found that treatment with silibinin not only obviously reduced ATP level, but also depleted glucose-6-phosphate and pyruvate and downregulated the protein levels of HK II, PFKP, and PKM2. This suggested that silibinin induced energy failure in glioma cells via suppressing glycolysis, which was also supported by the study showing that silibinin suppressed glycolytic activity in pancreatic cancer cells^29^. Although we did not further investigate whether AMPK/mTOR pathway was activated by silibinin, AMPK/mTOR activation was reported to account for silibinin-triggered autophagic death in renal carcinoma cells^23^. Thus, we thought that silibinin induced lethal autophagy in glioma cells via suppression of glycolysis.

Despite it was generally accepted that the protection of autophagy against cell death is associated with inhibition of oxidative stress by clearing damaged mitochondria^5^, several studies have shown that autophagy plays a role in promotion of oxidative stress. Autophagy was reported to improve intracellular ROS levels via selective degradation of catalase, which is an enzyme responsible for clearing hydrogen peroxide^30^. Moreover, it was also found that HK II which accounts for generating glucose-6-phosphate during the process of glycolysis is also a selective substrate of autophagy^31^. Notably, knockdown of HK II with SiRNA was confirmed to cause accumulation of intracellular ROS^32^. Thus, these studies revealed that autophagy also plays an important role in improving intracellular ROS levels. Accumulating evidences showed as well that oxidative stress was responsible for silibinin-induced death in various types of cancer cells^8,12,33^. Recent studies proved that activation of p53 is a primary pathway via which silibinin improved intracellular ROS levels. Activated p53 promoted silibinin-induced oxidative stress mainly through two pathways, one is triggering excessive generation of ROS in mitochondria by activation of JNK, and the other is causing depletion of antioxidant GSH^34,35^. Of note, activated p53 was reported to deplete GSH via negative regulation of cysteine level and xCT expression^21^. In this study, we found that blocking autophagy with 3MA or bafilomycin A1 not only inhibited silibinin-induced phosphorylation of p53, but also prevented silibinin-triggered downregulation of xCT, depletion of cysteine and GSH and improvement of hydrogen peroxide. Although we did not investigate in this study the role of autophagy in silibinin-induced activation of p53, it was reported that autophagy promoted p53 activation via selectively degradation of Δ133p53α (an inhibitory p53 isoform that can inhibit full-length p53)^36^. Therefore, our data suggested that autophagy contributed to silibinin-induced oxidative stress in glioma cells via promotion of p53 activation. Additionally, it was also reported that glycolysis was inhibited significantly when glioma cells were treated with hydrogen peroxide^20^. In this study, we found that the inhibitory effect of silibinin on glycolysis was apparently reversed when hydrogen peroxide was suppressed by supplement of GSH. Therefore, silibinin also induced a positive feedback between glycolysis dysfunction and autophagy, which resulted in excessive activation of autophagy.

Autophagy was reported to account for the glioma cell death induced by ceremide or compound AT101 via excessively removing mitochondria^10,11^, whereas accumulating evidences have shown that only damaged mitochondria, which are separated from mitochondrial network could be recognized, encapsulated and then degraded via autophagy pathway^37^. Thus, mitochondria damage is prior to its removal by autophagy. It has been demonstrated that apoptosis, necroptosis, and parthanatos contributed to cell death via causing mitochondrial damage and nuclear translocation of AIF^38–40^, but it remains elusive whether autophagy plays a role in impairing mitochondria and promoting AIF translocation from mitochondria to nuclei. In this study, we found that blocking autophagy with 3MA, bafilomycin A1 or by knocking down ATG5 with SiRNA not only inhibited silibinin-induced glioma cell death, but also prevented mitochondrial accumulation of superoxide and nuclear translocation of AIF. As a protein located within the space between mitochondria outer membrane and inner membrane, AIF is only released from damaged mitochondria, which had depleted membrane potential^41,42^. Within nucleus, AIF acts as a nuclease after cooperating with cyclophilin A to form a DNA-degrading complex at the location of DNA double strand breaks, which eventually leads to irreversible chromatinolysis and cell death^41,42^. Consistent with previous report showing that silibinin induced glioma cell death via promotion of AIF translocation from mitochondria to nuclei^15^, our data in this study proved that knockdown of AIF with SiRNA not only prevented silibinin-induced its accumulation in nuclei, but also inhibited glioma cell death. Thus, autophagy contributes to silibinin-induced glioma cell death via causing mitochondria damage and AIF translocation form mitochondria to nuclei. Previously, intracellular ROS were found to play a crucial role in promotion of mitochondria damage and AIF translocation from mitochondria to nuclei. It was reported that treatment with hydrogen peroxide obviously triggered mitochondrial depolarization and nuclear translocation of AIF in glioma cells^40^. Therefore, these studies suggested that improvement of intracellular hydrogen peroxide is a pathway via which autophagy promoted silibinin-induced mitochondrial damage.

As a protein targeting mitochondria, BNIP3 is excessively expressed in human gliomas^43^. BNIP3 plays dual roles in regulation of cell demise. Suppression of BNIP3 by upregulation of miR-145 was reported to induce apoptosis in glioma cells^43^. However, BNIP3 upregulation could also lead to cell death. It was reported that overexpressed BNIP3 contributed to baicalein-induced apoptosis in osteosarcoma cells, hydrogen peroxide-induced autophagic death in glioma cells, and doxorubicin-induced necrosis in cardiac myocytes^44,45^. As well as Bax, Bim and Noxa which induce mitochondrial dysfunction upon accumulation on mitochondria^46–48^, BNIP3 was confirmed to trigger MPTP opening and loss of mitochondrial membrane potentials when its C-terminal transmembrane domain is inserted into mitochondrial outer membrane^10^. Jiang et al. proved that BNIP3 was involved in regulation of silibinin-induced reduction of mitochondrial membrane potentials in human breast cancer cells^7^. Consistently, we found in this study that silibinin not only upregulated the protein level of BNIP3, but also promoted BNIP3 translocate to mitochondria. In contrast, knockdown of BNIP3 with SiRNA obviously inhibited silibinin-induced mitochondrial depolarization, accumulation of mitochondrial superoxide and nuclear translocation of AIF, as well as rescued glioma cell death. Thus, BNIP3 contributed to silibinin-induced glioma cell death via causing mitochondrial damage. Although HIF-1α is a transcription factor regulating BNIP3 expression^49^, we found in this study that silibinin treatment resulted in time-dependent downregulation of HIF-1α. This was also supported by previous study showing silibinin inhibited HIF-1α expression in prostate cancer cells^14^. Oxidative stress was also reported to contribute to baicalein-induced BNIP3 upregulation in human osteosarcoma cells^44^. Consistently, we found as well that silibinin-induced BNIP3 upregulation and translocation to mitochondria were both apparently reversed when hydrogen peroxide was suppressed by supplement of GSH or autophagy was blocked. Given that silibinin-induced improvement of hydrogen peroxide was inhibited in the presence of 3MA or bafilomycin A1, we think that autophagy promoted silibinin-induced damage in mitochondria via regulation of BNIP3.

In conclusion, we demonstrated in this study that silibinin activates lethal autophagy though suppression of glycolysis. Autophagy improves intracellular hydrogen peroxide via depletion of GSH and cysteine by promoting p53 phosphorylation. Ultimately, hydrogen peroxide triggers BNIP3-dependnet mitochondria damages and nuclear translocation of AIF. Therefore, autophagy contributes to silibinin-induced glioma cell death via causing mitochondrial damage and nuclear translocation of AIF.

## Supplementary information

Legends for supplementary figures

Figure S1

Figure S2

Figure S3

Figure S4

Figure S5

Figure S6

Figure S7

## Data Availability

The data supporting the findings of this study are available within the article and supplementary files or from the authors upon reasonable request.
