# Supplementary material for: Autophagy activated by silibinin contributes to glioma cell death via induction of oxidative stress-mediated BNIP3-dependent nuclear translocation of AIF
Source: Cell Death Dis. 2020 Aug 14;11(8):630. doi: 10.1038/s41419-020-02866-3 (PMC7429844; doi:10.1038/s41419-020-02866-3)
Supplement: Supplementary file 1 — Legends for supplementary figures [file 41419_2020_2866_MOESM1_ESM.doc]

**Legends for supplementary figures**

**Figure S1 Statistical analysis of the quantified bands of western blotting in Figure 1, and LDH release assay of the effects of necroptosis inhibitors and ferroptosis inhibitors on silibinin-induced glioma cell death.**

(A-C) Statistical analysis of the quantified bands of western blotting showed that silibinin induced time-dependent upregulation of ATG5, LC3 II and p62(SQSTM1). (D) Silibinin-induced upregulation of LC3 II was inhibited by 3MA, but was enhanced by bafilomycin A1. (E) The downregulation of p62(SQSTM1) caused by silibinin was reversed in the presence of 3MA or bafilomycin A1. (F)Silibinin-induced upregulation of ATG5 was inhibited in the cells transfected with ATG5 SiRNA. (G)Knockdown of ATG5 with SiRNA prevented silibinin-induced upregulation of LC3 II. (H)Silibinin-induced downregulation of p62(SQSTM1) was significantly reversed when ATG5 was knocked down with SiRNA. (I) LDH release assay showed that necroptosis inhibitor Nec-1, GSK-872 or NSA did not prevent silibinin-induced glioma cell death. (J) LDH release assay showed that ferroptosis inhibitor deferoxamine or fer-1 did not inhibit silibinin-induced glioma cell death. *: *p* < 0.01 versus control group. The values are expressed as mean±SEM (n=5 per group).

**Figure S2 Statistical analysis of the reduction of red fluorescence detected by flow cytometry combined with JC-1 staining, and the quantified bands of western blotting in Figure 2.**

(A) Statistical analysis of the reduction of red fluorescence detected by flow cytometry combined with JC-1 staining showed that silibinin induced time-dependent reduction in the red fluorescence. (B) Silibinin significantly induced time-dependent decrease of AIF in mitochondrial fractions. (C) Silibinin markedly improved cytoplasmic level of AIF in a time-dependent manner. (D) Silibinin treatment resulted in time-dependent improvement of AIF in nuclear fractions. (E) Mitochondrial level of AIF reduced in the cells transfected with AIF SiRNA, which further decreased in the presence of silibinin. (F) Knockdown of AIF with SiRNA inhibited silibinin-induced improvement of AIF in cytoplasmic fractions. (G) Silibinin-induced increase of AIF in nuclear fractions was prevented when AIF was knocked down with SiRNA. (H) The decrease of AIF in mitochondrial fraction caused by silibinin was inhibited in the presence of 3MA or bafilomycin A1. (I) The increase of AIF in cytoplasmic fraction induced by silibinin was prevented when the cells were pretreated with 3MA or bafilomycin A1. (J) The improvement of AIF in nuclear fraction due to silibinin treatment was abrogated by pretreatment with 3MA or bafilomycin A1. (K) Silibinin-induced reduction of AIF in mitochondrial fraction was inhibited in the cells transfected with ATG5 SiRNA. (L) Knockdown of ATG5 with SiRNA prevented silibinin-induced increase of AIF in cytoplasmic fraction. (M) Silibinin-induced improvement of AIF in nuclear fractions was abrogated when ATG5 was knocked down with SiRNA. *: *p* < 0.01 versus control group. The values are expressed as mean±SEM (n=5 per group).

**Figure S3. Statistical analysis of the quantified bands of western blotting in Figure 3.**

(A) Silibinin upregulated BNIP3 expression in a time-dependent manner. (B) Silibinin induced time-dependent improvement of BNIP3 in mitochondrial fractions. (C) Silibinin-induced upregulation of BNIP3 was prevented in the cells transfected with BNIP3 SiRNA. (D) Knockdown of BNIP3 with SiRNA prevented silibinin-induced improvement of BNIP3 in mitochondrial fractions. (E) Knockdown BNP3 with SiRNA inhibited silibinin-induced reduction of AIF in mitochondrial fractions. (F) Silibinin-induced improvement of AIF in cytopalsmic fractions was prevented when BNIP3 was knocked down with SiRNA. (G) Knockdown BNP3 with SiRNA abrogated silibinin-induced increase of AIF in nuclear fractions. (H) Silibinin-induced upregulation of BNIP3 was inhibited by pretreatment with 3MA or bafilomycin A1. (I) Silibinin-induced improvement of BNIP3 in mitochondrial fractions was inhibited in the presence of 3MA or bafilomycin A1. *: *p* < 0.01 versus control group. The values are expressed as mean±SEM (n=5 per group).

**Figure S4. Statistical analysis of the quantified bands of western blotting in Figure 4, and the effect of GKT137831 on silibinin-induced hydrogen peroxide and glioma cell death.**

(A) Silibinin induced time-dependent downregulation of HIF-1α in nuclear fractions. (B) LDH release assay showed that pretreatment with GSK137831 at 500μmol/L for 1h significantly inhibited silibinin-induced glioma cell death. (C) H_2_O_2_ assay showed silibinin-triggered improvement of H_2_O_2_ was prevented in the presence of GSK137831. (D) Silibinin induced time-dependent upregulation of GPX4 in glioma cells. (E) Pretreatment with GSH prevented silibinin-induced BNIP3 over-expression. (F) Silibinin-induced BNIP3 upregulation in mitochondrial fractions was inhibited by GSH. (G) H_2_O_2_ alone induced BNIP3 upregulation in a time-dependent manner. (H) H_2_O_2_ alone triggered time-dependent improvement of BNIP3 in mitochondrial fractions. *: *p* < 0.01 versus control group. The values are expressed as mean±SEM (n=5 per group).

**Figure S5. Statistical analysis of the quantified bands of western blotting in Figure 5.**

(A) Silibinin induced time-dependent downregulation of xCT. (B) Silibinin triggered p53 upregulation in a time-dependent manner. (C) Silibinin time-dependently improved the level of phospho-p53. (D) Silibinin-induced downregulation of xCT was prevented by pretreatment with 3MA or bafilomycin A1. (E) Silibinin-induced upregulation of p53 was inhibited in the presence of 3MA or bafilomycin A1. (F) Silibinin-triggered improvement of phospho-p53 was abrogated by 3MA or bafilomycin A1. *: *p* < 0.01 versus control group. The values are expressed as mean±SEM (n=5 per group).

**Figure S6. Statistical analysis of the quantified bands of western blotting in Figure 6.**

(A) Silibinin induced time-dependent downregulation of HK II. (B) Silibinin triggered PFKP downregulation in a time-dependent manner. (C) Silibinin time-dependently decreased the level of PKM2. (D) Silibinin-induced downregulation of HK II was prevented by pretreatment with GSH. (E) Silibinin-induced reduction of PFKP was inhibited by GSH. (F) Silibinin-triggered decrease of phospho-p53 was abrogated in the presence of GSH. (G) Silibinin-induced upregulation of ATG5 was prevented by pretreatment with GSH. (H) Silibinin-induced increase of LC3II was prevented by GSH. (I) Silibinin-induced downregulation of p62(SQSTM1) was prevented in the presence of GSH. *: *p* < 0.01 versus control group. The values are expressed as mean±SEM (n=5 per group).

**Figure S7. Statistical analysis of the quantified bands of western blotting in Figure 7.**

(A) Silibinin induced upregulation of ATG5 and LC3-II, but downregulation of p62. (B) Silibinin treatment resulted in downregulation of xCT, but upregulation of p53 and phospho-p53. (C) Silibinin triggered BNIP3 upregulation and accumulation on mitochondria. (D) Silibinin promoted AIF translocation from mitochondria to nuclei. (E) Silibinin treatment resulted in downregulation of HK II, PFKP and PKM2. *: *p* < 0.01 versus control group. The values are expressed as mean±SEM (n=5 per group).
