# Supplementary figures and images for: Autophagy activated by silibinin contributes to glioma cell death via induction of oxidative stress-mediated BNIP3-dependent nuclear translocation of AIF
Source: Cell Death Dis. 2020 Aug 14;11(8):630. doi: 10.1038/s41419-020-02866-3 (PMC7429844; doi:10.1038/s41419-020-02866-3)

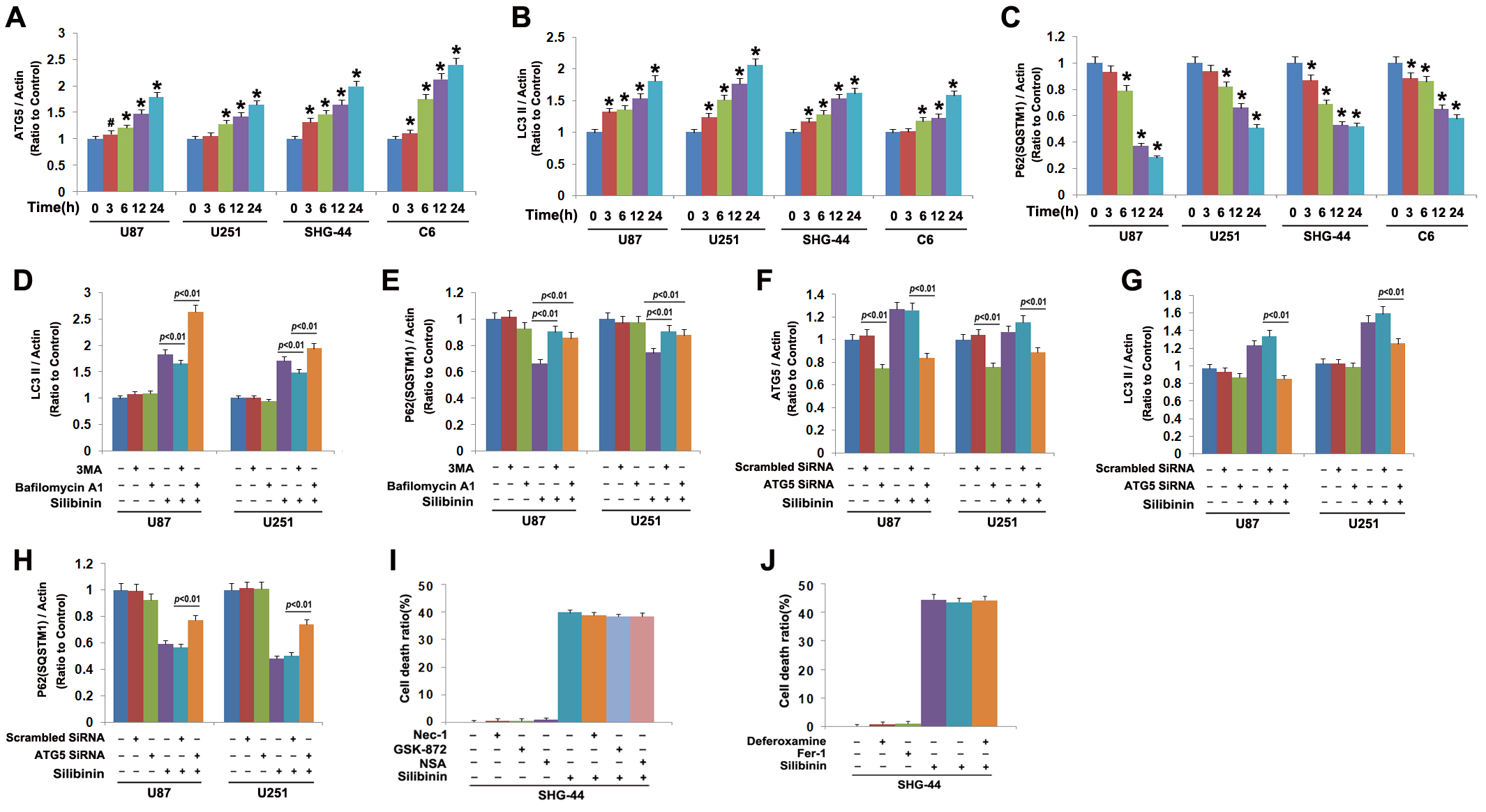

Supplement: Supplementary file 2 — Figure S1 [file 41419_2020_2866_MOESM2_ESM.jpg]

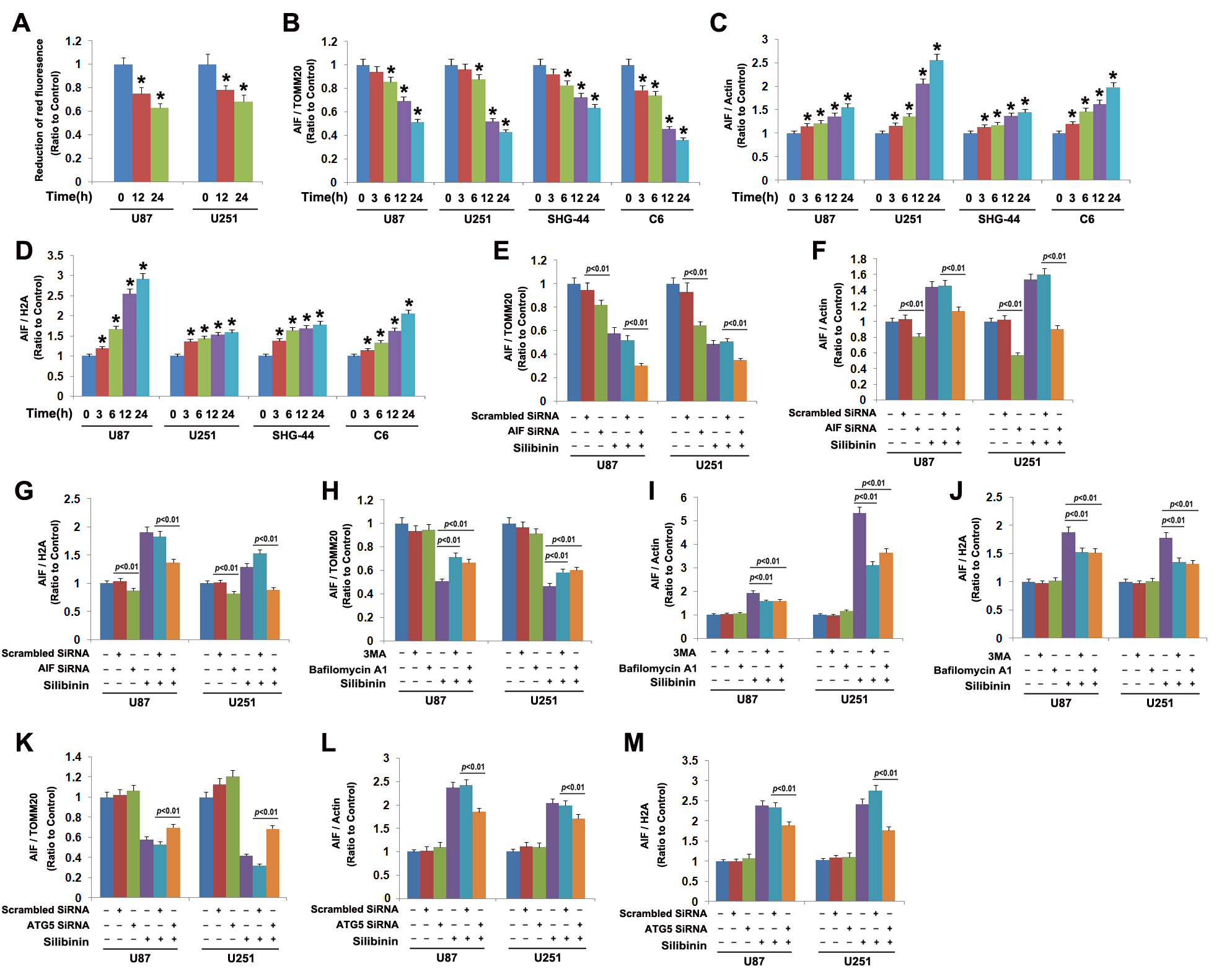

Supplement: Supplementary file 3 — Figure S2 [file 41419_2020_2866_MOESM3_ESM.jpg]

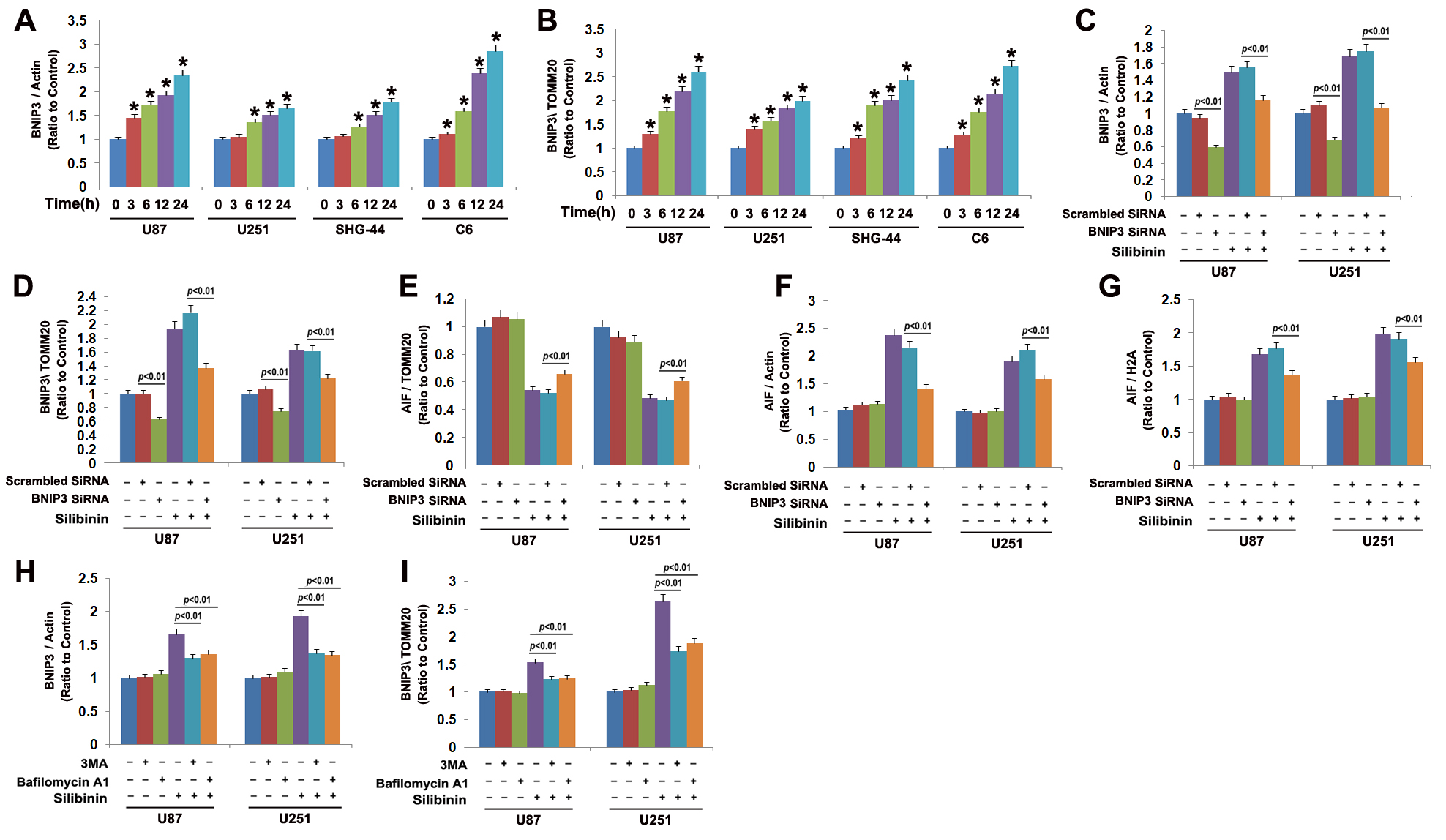

Supplement: Supplementary file 4 — Figure S3 [file 41419_2020_2866_MOESM4_ESM.jpg]

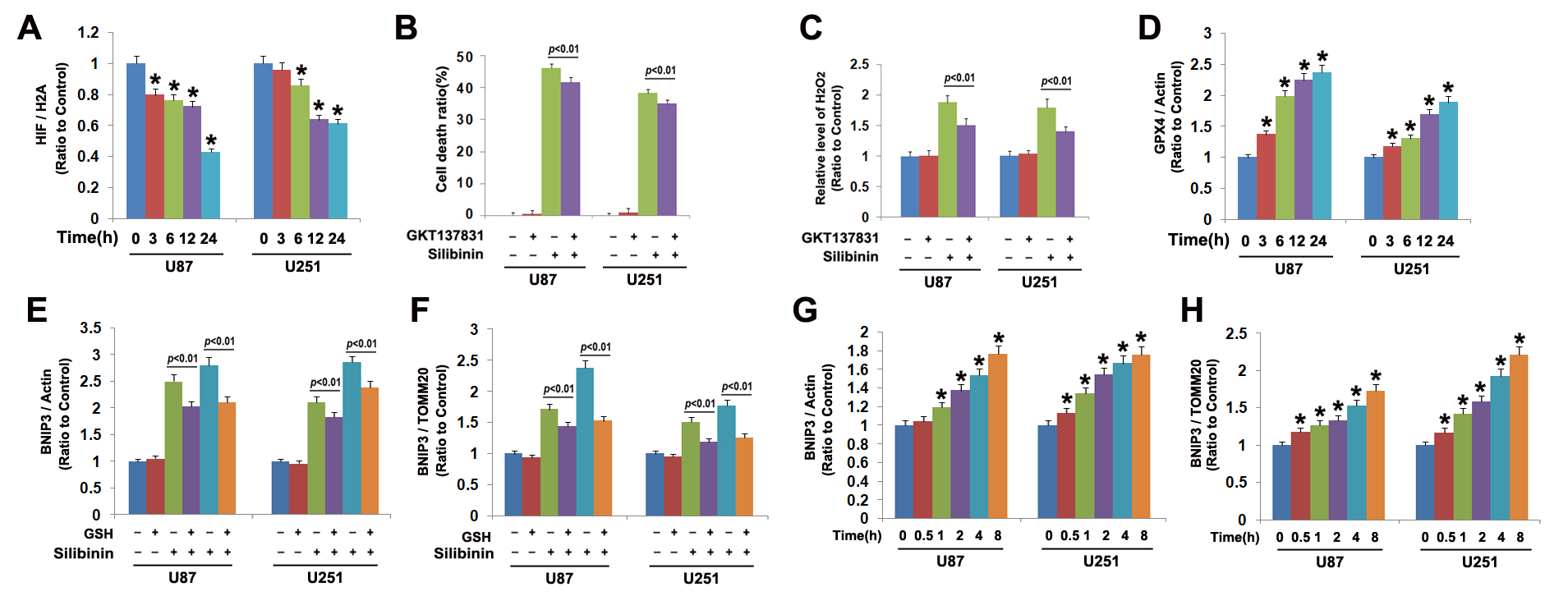

Supplement: Supplementary file 5 — Figure S4 [file 41419_2020_2866_MOESM5_ESM.jpg]

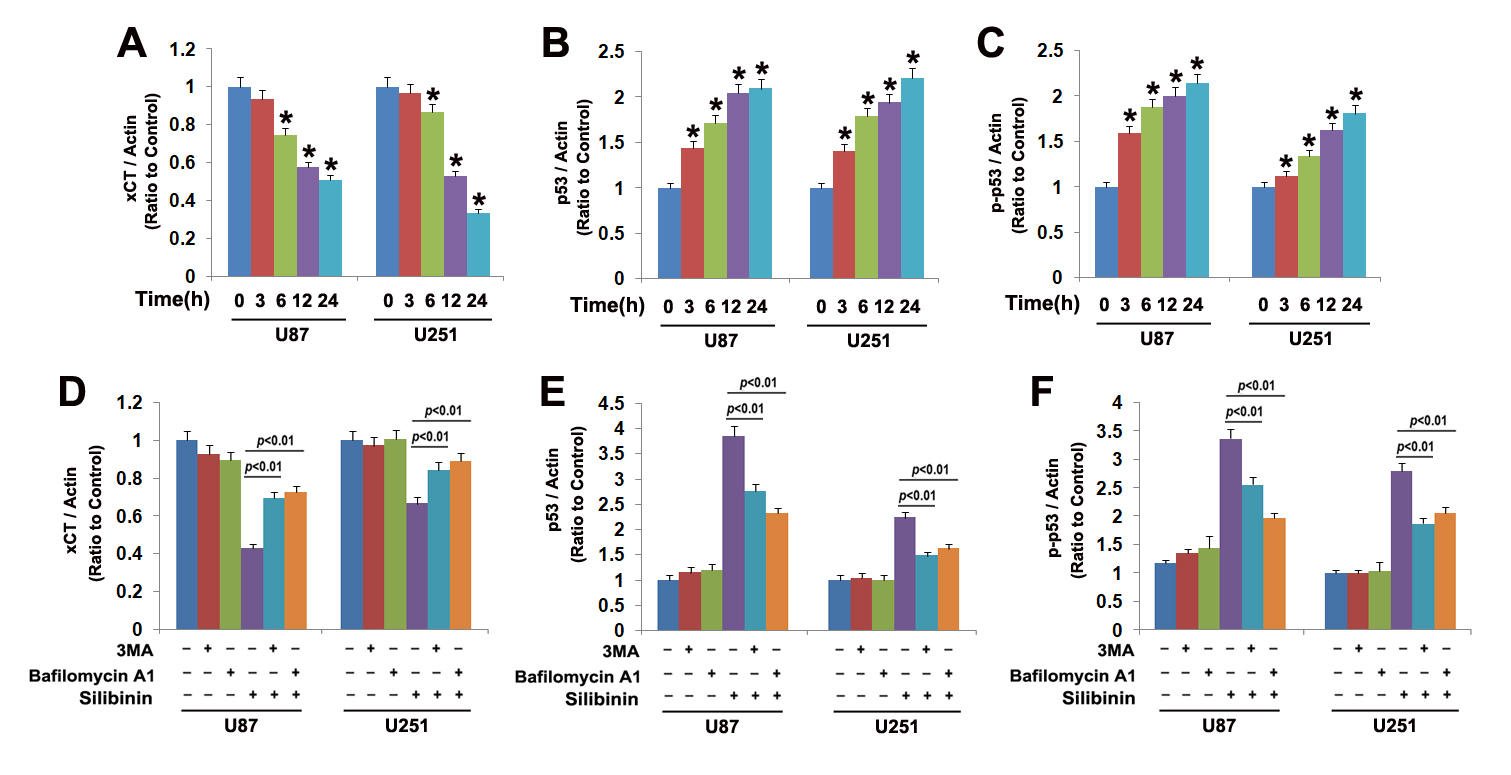

Supplement: Supplementary file 6 — Figure S5 [file 41419_2020_2866_MOESM6_ESM.jpg]

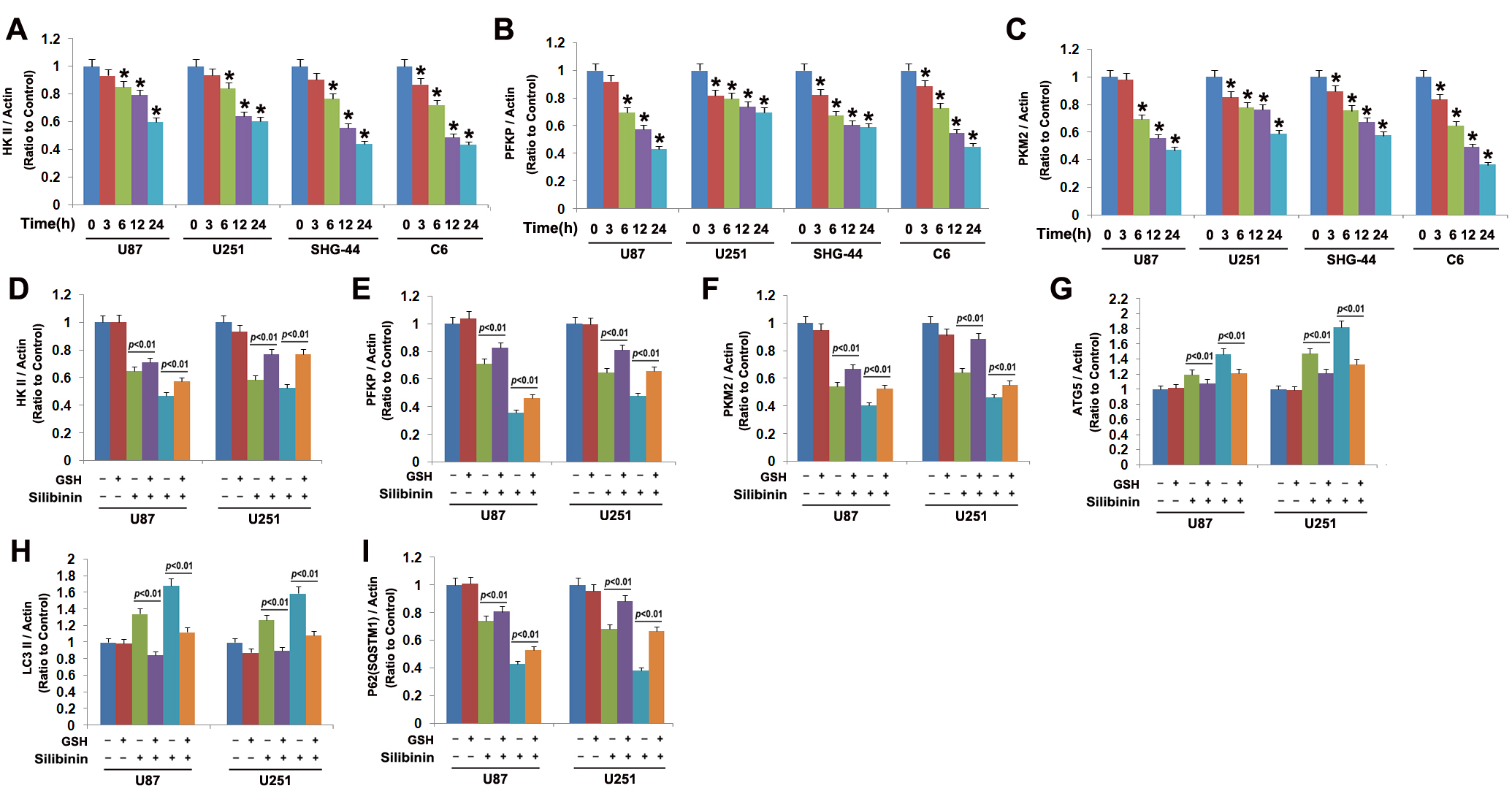

Supplement: Supplementary file 7 — Figure S6 [file 41419_2020_2866_MOESM7_ESM.jpg]

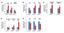

Supplement: Supplementary file 8 — Figure S7 [file 41419_2020_2866_MOESM8_ESM.jpg]
